# Equivalent Circuit Extraction of SAW Resonator with Spurious Modes Interference over a −55 °C to 85 °C Temperature Range

**DOI:** 10.3390/mi17080893

**Published:** 2026-07-25

**Authors:** Xianli Tang, Yonghao Jia, Yuandong Gu

**Affiliations:** 1 School of Microelectronics, Shanghai University, Shanghai 200444, China; tangxl1229@gmail.com; 2 State Key Laboratory of Millimeter Waves, School of Information Science and Engineering, Southeast University, Nanjing 210096, China

**Keywords:** admittance parameters, equivalent circuit model, filter, surface acoustic wave, scattering parameter measurements, spurious modes, resonators, temperature

## Abstract

Surface acoustic wave (SAW) resonators are widely employed to design radio-frequency (RF) filters in wireless communication. To adapt to various application scenarios, the article proposes an equivalent circuit model based on the Butterworth–Van Dyke (BVD) model for SAW devices operating with spurious modes and at extreme ambient temperatures. In cases where the resonance frequencies of the spurious modes are close to those of the main mode, isolation capacitances (IC) are proposed in the modeling process. With the IC, the different resonance frequencies produced by the proposed equivalent circuit model can be flexibly adjusted. The extreme temperature influence on the SAW resonators is investigated using the proposed model. In the temperature-dependent test environment, the performance of the SAW devices changes, and these changes are captured by the proposed model. Especially for the resonators’ spurious-mode frequencies, which are less influenced by temperature near room temperature unless extreme temperatures are applied. The parameters motional resistance *R_m_* and motional inductance *L_m_* are considered temperature-dependent and are used to describe the influence of the ambient temperature. The RF characteristics of the SAW devices are modeled with the proposed model and verified with measurement data. The consistent results between the measured data and simulated data indicate that the proposed model is accurate and the modeling work is effective.

## 1. Introduction

While fifth-generation (5G) communication systems have achieved large-scale commercialization, sixth-generation (6G) is already emerging as the next frontier, driving the demand for extensive high-performance filters to meet the stringent requirements of emerging applications [1,2,3,4,5]. Acoustic wave (AW) filters [6,7] have been widely employed in wireless communication due to their small size. Besides this merit, its own skirt selectivity, low insertion loss (IL), and compact size make it suitable for employment in the RF front of wireless communication systems [8,9]. Due to its massive application scenarios and rapidly increasing number of devices even in a mobile phone [10], the reliability [11,12] requirement of the AW filter becomes increasingly significant. One of the most concerning issues is the thermal influence on the acoustic wave resonators (AWRs) [13,14,15]. Usually, this device characteristic can be investigated by a device thermal modeling process [10,14,16,17]. Its benefit is that the AWR device with spurious modes can be analyzed with the model, and the AW filter can also be designed with this device model.

The performance of the AWR is significantly affected by thermal effects arising from two primary sources: self-heating [10] induced by high input radio-frequency (RF) power and ambient temperatures in the device’s operating environment. In typical cases, transmitter filters are subjected to high power levels [14], where a portion of the applied power is dissipated as heat, causing the filter to heat up. In turn, this temperature increase changes the filter’s RF performance. Moreover, the self-heating may drastically reduce device reliability. In another case of low-power applications, surface acoustic wave (SAW) filters exhibit lower self-heating effects but require further study. They are still affected by extreme ambient temperatures since they operate on the principle of electromechanical transduction. When RF Power is applied, the transducer at the input terminal (e.g., interdigitated electrodes) converts the RF signal into mechanical vibrations that propagate through the piezoelectric material. In turn, the transducer at the output terminal reconverts these acoustic waves back into an RF signal [16]. The process is significantly affected by ambient temperature, and the temperature coefficient of frequency (TCF) is typically used to characterize this influence. The parameters are widely investigated [18,19,20,21,22] and used to quantify the frequency drift of SAW filters with temperature variations, typically expressed in ppm/°C. Most materials become softer as temperature increases. Thus, the filter passbands of RF AW devices shift downward as temperature rises [10], indicating a negative TCF. To mitigate this adverse effect and enhance thermal stability, engineering strategies such as incorporating a temperature-compensation layer [14] or adopting composite substrate structures are commonly implemented to counteract the inherent negative TCF [10] and achieve a near-zero overall frequency drift over a wide operational temperature range [18]. It is usually called TC-SAW, in which the device designs incorporate SiO_2_ compensation layers to reduce TCF and ensure stable filter performance across wide temperature ranges [17]. Based on the above, a non-monotonic change in the resonance frequency of the SAW resonators is observed [23], as shown in the following paper. Given that the resonators may exhibit spurious modes [10], the effect of extreme temperatures on the SAW device with spurious modes needs to be studied.

In response to this challenge, various methodologies have been developed over recent years to enable AW filter and multiplexer designers to evaluate and optimize SAW devices under diverse conditions. For the modeling approaches of the SAW devices, a lot of work with the Finite Element Method (FEM) has been reported [24,25,26], which is employed to investigate the resonator’s performance, such as the electromechanical coupling factor (*k*^2^), *Q*-factor, and spurious modes [10]. Moreover, the 3-D FEM simulation method has been employed to obtain accurate results [14,15,27,28,29,30]. However, the thermal 3D FEM method is not suitable for SAW filter circuit designs due to its long computation time. For SAW devices, the P-matrix approach and the coupling-of-modes (COM) model have been established as the predominant modeling tools, providing fast and accurate simulations when spurious modes are well suppressed [10]. In the end, these models seem time-consuming to implement filter designs in software such as Advanced Design System (ADS) and Simulation Program with Integrated Circuit Emphasis (SPICE).

Due to the transducer structures and the distributed-parameter nature of these SAW devices, it is necessary to improve the efficiency of filter designs by integrating fabricated SAW devices into external passive networks or active RF front-end circuits. In the following, the equivalent circuit model (ECM) and its related behavioral model [16,23,31,32,33], based on lumped elements, offer a more practical alternative for circuit designs. Among these, the Butterworth–Van Dyke (BVD) model and its modified variants (MBVD) have emerged as the industry-standard model for describing the electrical behavior of SAW resonators. By representing the resonator as a parallel combination of a static capacitance and a series RLC branch, the BVD model not only captures the primary resonance characteristics but also integrates seamlessly with standard circuit simulators (e.g., ADS) for RF circuit designs. For the thermal influence on the SAW devices, the electro-thermal ECMs of SAW devices are provided [14,16,17,23,34]. In [14,17], the implementation of a temperature-dependent inductance, *L_m_*(T), in the MBVD model is proposed to account for the shift in the frequency response due to TCF. In [16,23,33], the temperature-dependent *L_m_*(T), *C_m_*(T), *R_m_*(T), and *C*_0_(T) are investigated to model the SAW device under temperature influence.

In the article, unlike the previous excellent studies, the SAW resonators with spurious modes are investigated using the proposed model based on the BVD model and evaluated under temperature-dependent conditions. The proposed work is very useful for SAW devices and their SAW-based filters for use in extreme-temperature environments. The rest of the article is organized as follows. Section 2 describes the proposed modeling, which includes the proposed model and the influence of temperature on the model parameters. In Section 3, the proposed model of the SAW resonators is verified against the measurement data, and the discussion is also presented there. In the end, Section 4 concludes the paper.

## 2. Modeling of the Acoustic Surface Wave Resonators

### 2.1. Proposed Equivalent Circuit Model of the Acoustic Wave Resonators

**Figure 1:**
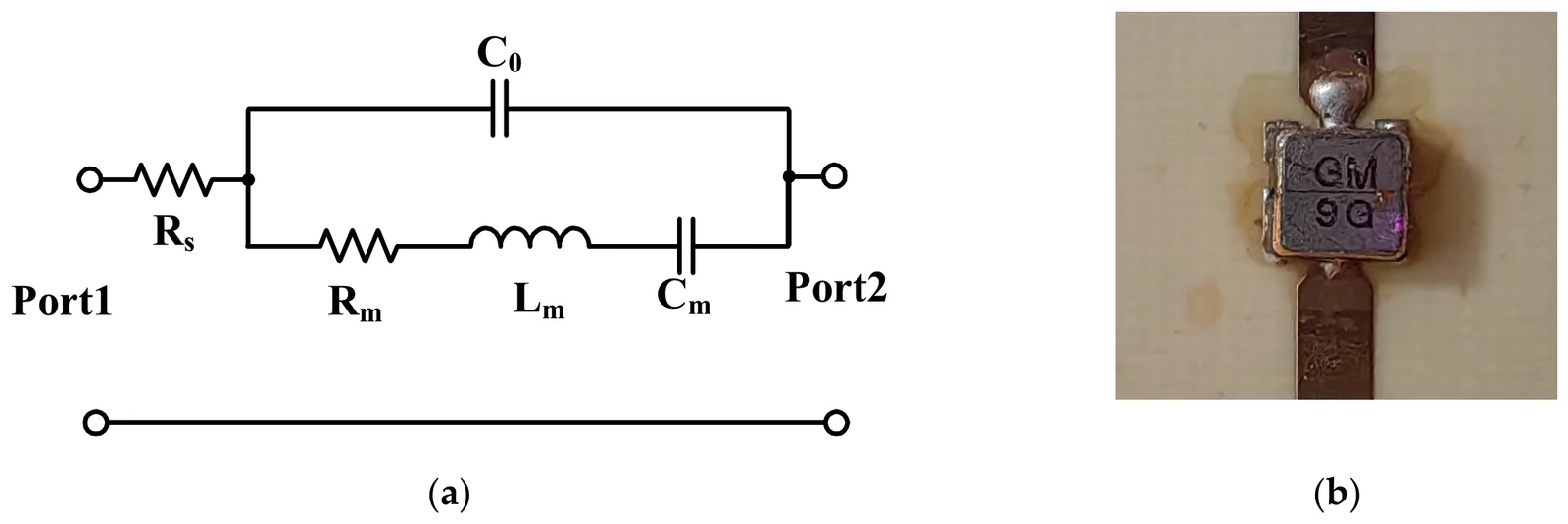
(**a**) Equivalent circuit model; (**b**) Commercial SAW resonator investigated.

To analyze the resonator performance of AWRs, many device models have been investigated [10]. Due to BVD’s simple structure and ease of parameter extraction, the BVD model has been widely investigated by researchers and is also included in the filter design software. Figure 1a shows the equivalent circuit model employed for SAW resonators without spurious modes.

**Figure 2:**
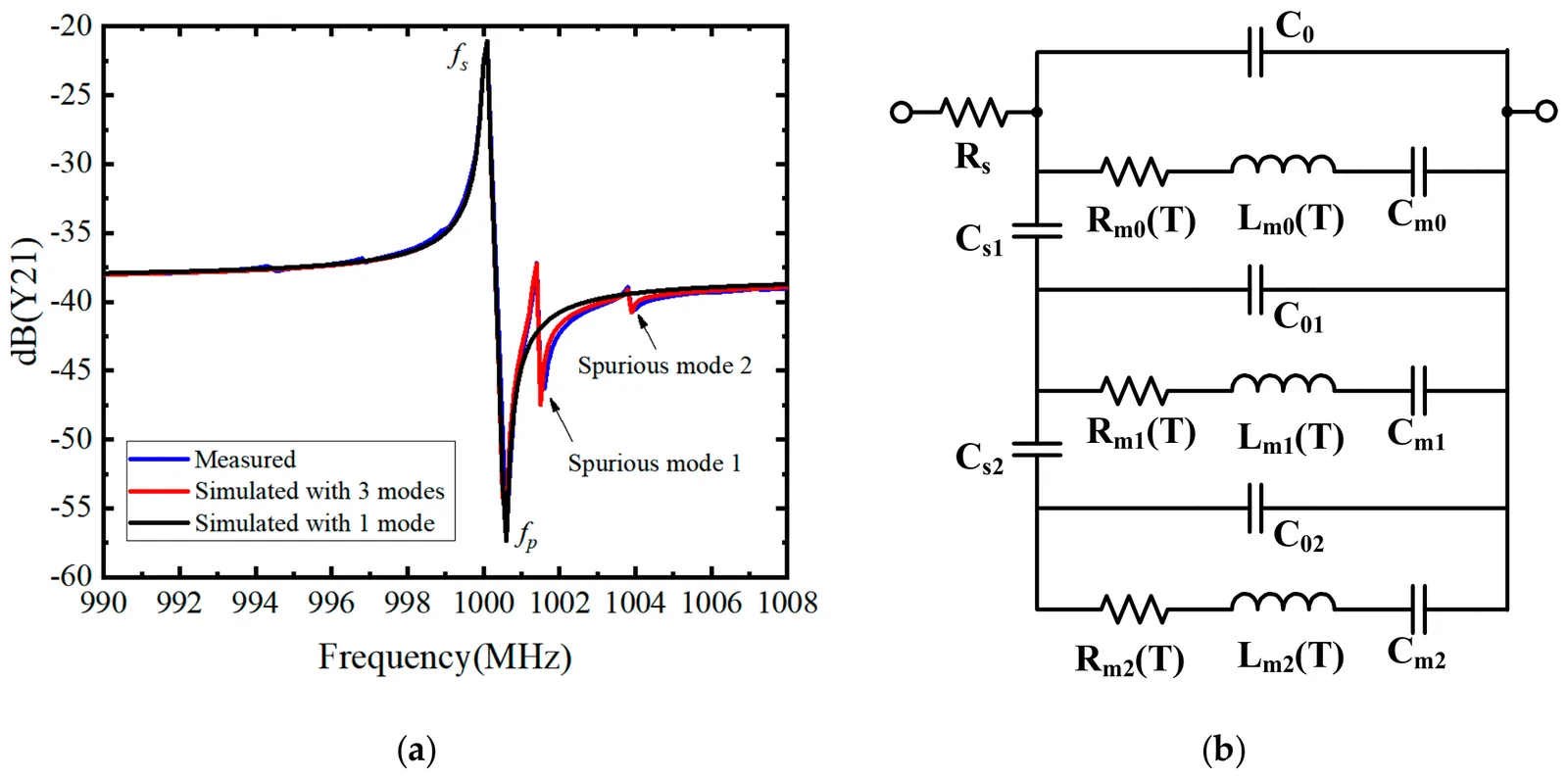
(**a**) Fitting of the Y parameters of the resonators; (**b**) Proposed model with spurious response based on the BVD model.

The model is composed of two parallel branches: one branch consists of the motional resistance *R_m_*, the motional inductance *L_m_*, and the motional capacitance *C_m_*, and the other branch consists of the parallel capacitance *C*_0_. The commercial SAW resonator is shown in Figure 1b, and the parameter values in its datasheet are given in Table 1. These parameter values are used to compare with the extracted parameters obtained using the traditional extraction method, which is based on an optimization process and the measurement data. The device studied in this work is an SMD-packaged SAW resonator produced by TAI-SAW (Taoyuan 324, Taiwan). Its manufacturer code is TC0528A, and its size is 3 mm × 3 mm × 1.4 mm. Its typical center frequency is 1000 MHz. The circuit in Figure 1a can be regarded as a two-port network when considering that it has a common ground. Thus, SAW devices are characterized by the admittance parameters, which represent the ratio of the port current and voltage of the SAW device. They have been widely employed to determine the resonator properties [10,23]. The Y_11_ parameter is the current divided by the voltage of port 1 when port 2 is short-circuited. The Y_22_ parameter is the current divided by the voltage of port 2 when port 1 is short-circuited. The Y_21_ parameter is the current at port 2 divided by the voltage at port 1 when port 2 is short-circuited. For this two-port network, the equivalent circuit consists only of a series path between the two ports. The input and output currents are equal in magnitude and opposite in direction. Therefore, the transfer admittances Y_21_ = Y_12_ = −Y_11_. The measured Y-parameter of the commercial SAW resonator is employed and shown in Figure 2a as the blue line. It includes three modes, a main mode and two spurious modes. The measured resonance frequency *f_s_* of the commercial SAW resonator is 999.84 MHz, and the anti-resonance frequency *f_p_* is 1000.47 MHz, which strictly conforms to the rule that zeros and poles alternate on the positive frequency axis. Based on the parameters of the model, the admittance can be calculated using traditional expressions. The starting values can be determined with the following expressions: *C*_0_ = *C*_01_ × ((*f_p_*/*f_s_*)^2^ − 1), *L*_m_ = 1/((2π*f_s_*)^2^*C*_m_). The parameters of *C*_01_ and *R_m_* are empirical and can be obtained with the optimization process. The calculated Y-parameter results are given in Expression (1). In the end, the extracted values of these parameters are provided in Table 1.

(1) $$Y_{21}={\left(R_{s}+{\left(j\omega C_{0}+\frac{1}{j\omega L_{m}+\frac{1}{j\omega C_{m}}+R_{m}}\right)}^{-1}\right)}^{-1}$$

where *ω* is the angular frequency.

The extracted parameter values and those provided by the supplier show some inconsistencies in Table 1. This may be due to the fitting results, as the simulation with 1 mode contains only the main mode in Figure 2a and neglects the spurious modes. The error occurs near the spurious mode frequency, where it accumulates and induces inconsistencies when optimized with minimum variance. To obtain more accurate fitting results, the spurious modes need to be carefully addressed. More parameters are considered when building the ECM of the SAW device with spurious mode responses. Those added parameters aim to produce more resonant frequencies with more branches. Therefore, the proposed model is shown in Figure 2b, which accounts for the spurious modes in Figure 2a. Based on the interest frequency bands, the main model with two spurious modes of larger amplitude is identified. To reduce the influence of each resonance frequency produced by the lumped elements, two isolation capacitances (ICs) *C_s_*_1_ and *C_s_*_2_ are proposed. The isolation capacitances are introduced to decouple the motional branches associated with the spurious modes from the main resonance, thereby preventing mutual interference between closely spaced resonant modes. Thus, three subcircuits for describing the resonances are shown in Figure 2b. With the proposed model with spurious modes and root-mean-square fitting methods [5,35,36], the accuracy results are shown in Figure 2a as the red line. According to the equivalent circuits in Figure 2a, the resonance frequencies can be obtained using the motional inductance *L_m_* and the motional capacitance *C_m_* [23]; they are given by Expression (2). The resonance frequency of the main mode can be obtained when *i* = 0. The resonance frequencies of spurious modes 1 and 2 can be obtained when *i* = 1 and *i* = 2, respectively. All parameter values are shown in Table 2. The value of *R*_s_ is 2.1 Ω.

(2) $$f_{si}=\frac{1}{2\pi \sqrt{L_{mi}C_{mi}}},i=0,1,2,$$

**Figure 3:**
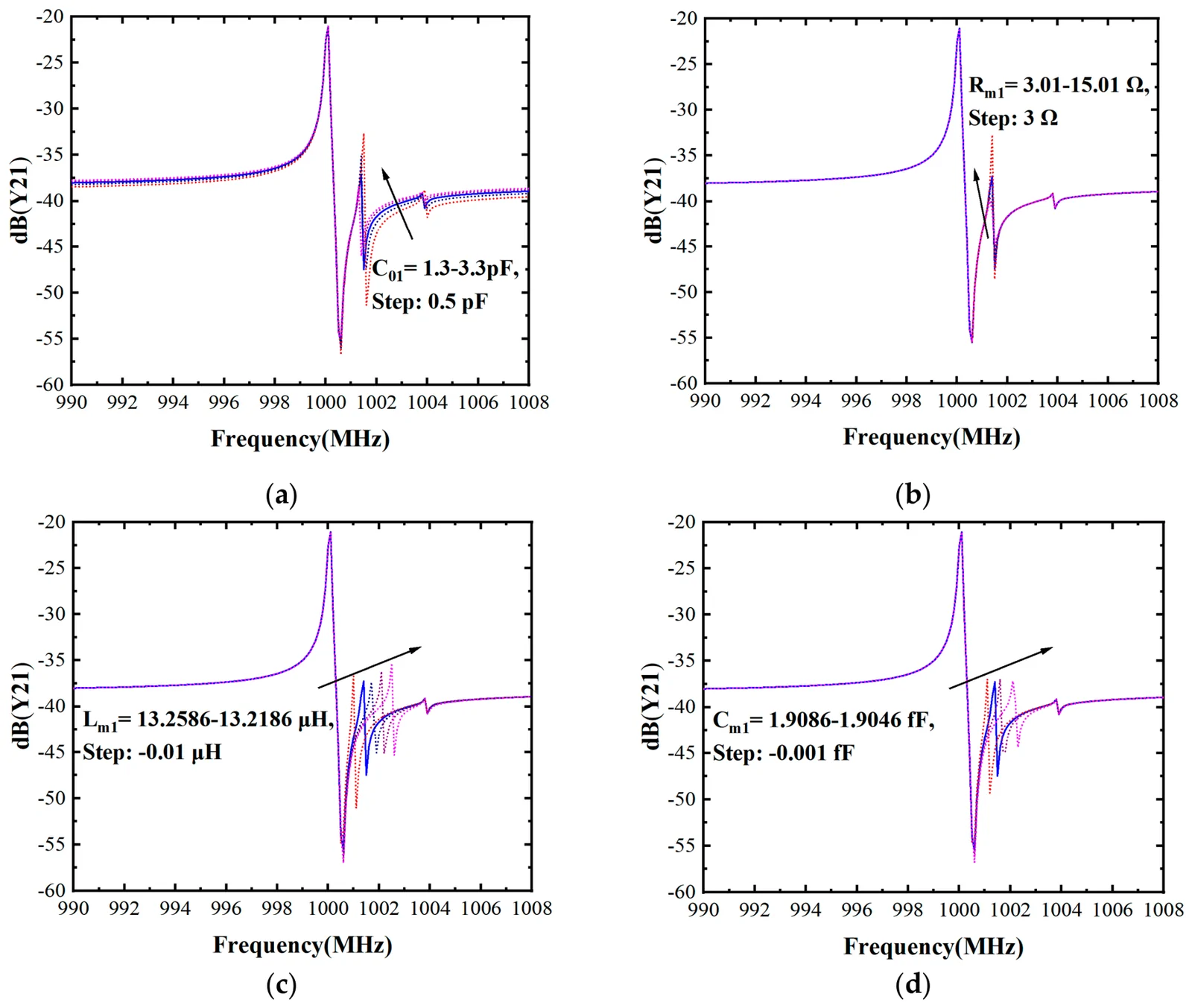
Main mode influenced by the parameters: (**a**) *C*_01_; (**b**) *R*_m1_; (**c**) *L*_m1_; (**d**) *C*_m1_ in the proposed model.

**Figure 4:**
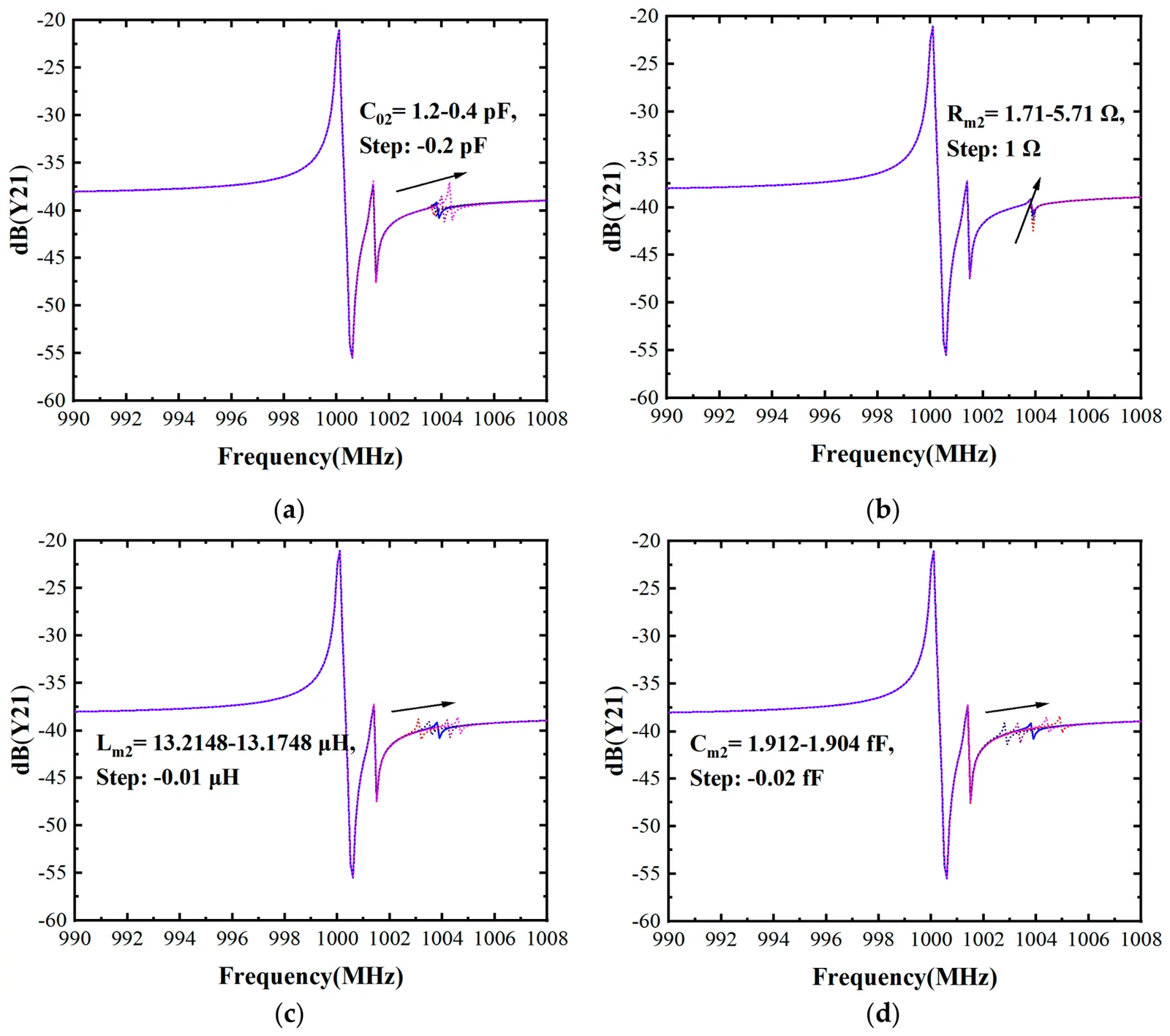
Main mode and spurious mode 1 influenced by the parameters: (**a**) *C*_02_; (**b**) *R*_m2_; (**c**) *L*_m2_; (**d**) *C*_m2_ in the proposed model.

Every subcircuit in Figure 2b consists of the motional resistance *R_m_*, motional inductance *L_m_*, and motional capacitance *C_m_*, and the other branch consists of the parallel capacitance *C*_0_. Unlike the reported papers [14,16,23,33,34], the two capacitances *C_s_*_1_ and *C_s_*_2_ are proposed to reduce the mutual influence between the subcircuits. Their values are 1 pF and 0.42 pF, and their benefits are shown in Figure 3 and Figure 4.

Based on the results in Figure 3 obtained using the proposed model in Figure 2b, the main mode is hardly affected by changes in *C*_01_, *R_m_*_1_, *L_m_*_1_, and *C_m_*_1_. Typically, the simulated spurious mode 1’s resonance amplitudes are mutually affected when the frequencies of the spurious mode and main mode are close. It differs from the practical characteristics of SAW resonators, which are indicated based on the measurement data in Figure 2a. With the proposed model using the capacitance C*_s_*_1_, the sharp resonance peak and the resonance amplitudes of the main mode are less influenced and can be described with the ECM. It is interesting and very useful, since the main mode is inevitably influenced when parameter values are changed in spurious modes, especially when the spurious mode frequencies and the main mode frequency are close. Both of them are near 1000 MHz. Sometimes it may be impossible to model the electrical performance of the SAWs when their frequencies are adjacent to each other, as shown in Figure 3. Only the parameter *C*_01_ has a slight effect on the main mode in the frequency band from 990 MHz to 998 MHz in Figure 3a. Its values range from 1.3 to 3.3 pF in 0.5 pF steps. Also, it has an influence on the spurious mode 2. Different from the parameter *C*_01_, the parameters *R_m_*_1_, *L_m_*_1_, and *C_m_*_1_ barely affect the main mode but only impact the spurious mode 1 within the same frequency band. Also, they have little influence on the spurious mode 2. In Figure 3b–d, the values of the parameters *R_m_*_1_, *L_m_*_1_, and *C_m_*_1_ are provided. For the influence of the IC *C_s2_*, the analysis is proposed as follows.

As shown in Figure 4, the main mode is hardly affected by changes in *C*_02_, *R_m_*_2_, *L_m_*_2_, and *C_m_*_2_, obtained with the proposed model in Figure 2b. Similarly, spurious mode 1 is unaffected by the parameters. The analysis is similar to that in Figure 3. It again indicates that the proposed model can be flexibly employed to describe the RF performance of SAWs that contain spurious modes. The frequencies of those modes are very close. In the same case, the main mode and spurious mode 1 are inevitably influenced when parameter values of *C*_02_, *R_m_*_2_, *L_m_*_2_, and *C_m_*_2_ are changed. Different from the results shown in Figure 3a, the parameter *C*_02_ has scarcely any effect on the main mode in the frequency band from 990 MHz to 998 MHz in Figure 4a. Its values range from 1.2 to 0.2 pF in −0.2 pF steps. In addition, it has hardly any influence on the spurious mode 1. As with parameter *C*_02_, the parameters *R_m_*_2_, *L_m_*_2_, and *C_m_*_2_ have little effect on the main mode and spurious mode 1 within the same frequency band. In Figure 4b–d, the values of the parameters *R_m_*_2_, *L_m_*_2_, and *C_m_*_2_ are provided.

### 2.2. Influence of the Temperature on the Parameters of the Acoustic Wave Resonators

For the investigated commercial SAW resonator in Figure 1b, the production datasheet shows that it has a negative temperature coefficient (TCF), and the value of its second-order temperature coefficient of frequency (TCF_2_) is −0.05 ppm/°C^2^. The TCF represents the temperature-dependent frequency drift, which is a critical performance-limiting factor for SAW resonators. In contrast, the temperature effects are deliberately exploited in thermoelectric sensors [37,38]. As described above, this drift is predominantly governed by the negative TCF intrinsic to the piezoelectric substrates in practical applications [10]. This pronounced frequency-temperature dependence poses a significant challenge for high-precision filter design and stable oscillator applications. The exceedingly low TCF_2_ exhibits outstanding linearity over the temperature range investigated. Such a linear characteristic is critical for practical RF filter applications, as it enables straightforward, accurate frequency compensation via a linear correction algorithm, thereby significantly simplifying the temperature-drift calibration process.

The good frequency-temperature performance is well-suited to the design of an RF filter. However, it is still unsuitable to implement the RF filter with an equivalent circuit model (ECM) for extreme operating environments. The parameters in the ECM are usually temperature-independent, as evidenced by the temperature-dependent resonance frequency and Expression (2). Thus, it is difficult to design an RF circuit with the ECM to employ in extreme temperature environments. To implement the device model in the design of an RF filter under different ambient temperature conditions, the influence of temperature on the parameters of the acoustic resonator model is investigated.

**Figure 5:**
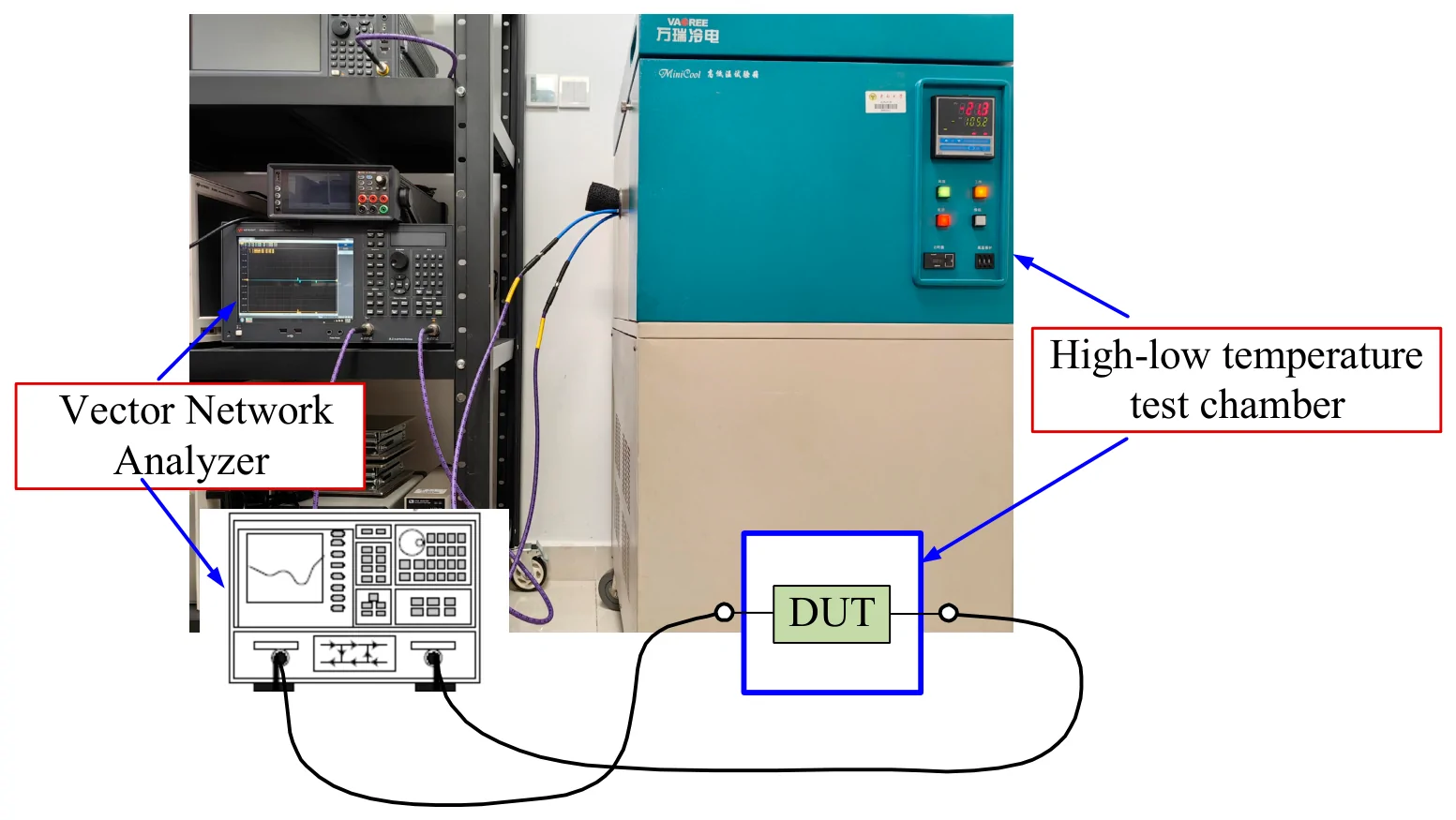
Experimental scenario of the influence of temperature on the SAW resonator.

The temperature-dependent RF measurements [35] are conducted to evaluate the temperature effect on the RF performance of the SAW device. The influence of temperature on the parameters of the ECM is first investigated. The temperature-dependent environmental test chamber is used in combination with the Vector Network Analyzer (Keysight E5071C, Keysight, Santa Rosa, CA, USA). The measurement setup is shown in Figure 5.

**Figure 6:**
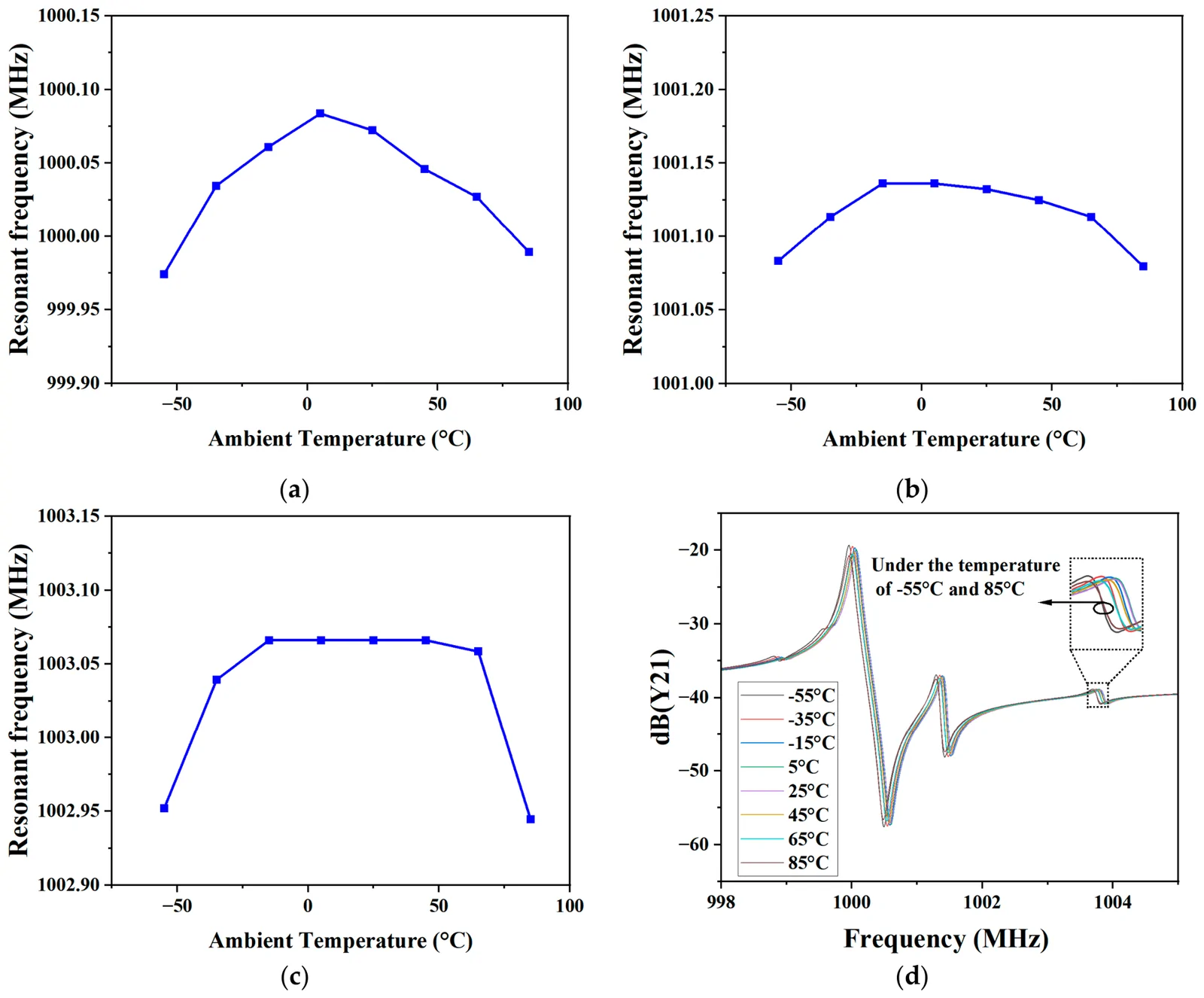
Temperature dependence of the resonant frequency. (**a**) main mode; (**b**) spurious mode 1; (**c**) spurious mode 2; (**d**) measurement data at −55 °C, −35 °C, −15 °C, 5 °C, 25 °C, −55 °C, and 85 °C.

According to Section 2.1, various parameters of the proposed model can be extracted. The values under extreme ambient environments are extracted in similar ways, which ensures building a proposed model under temperature-dependent conditions. This is because the extraction of the parameters of the model is based on an optimization process. Under different temperatures, the influence of temperature on the parameters of the acoustic resonators is considered. The test temperatures contain −55 °C, −35 °C, −15 °C, 5 °C, 25 °C, −55 °C, and 85 °C. According to Expression (2), the temperature dependence of the resonant frequencies is shown in Figure 6.

**Figure 7:**
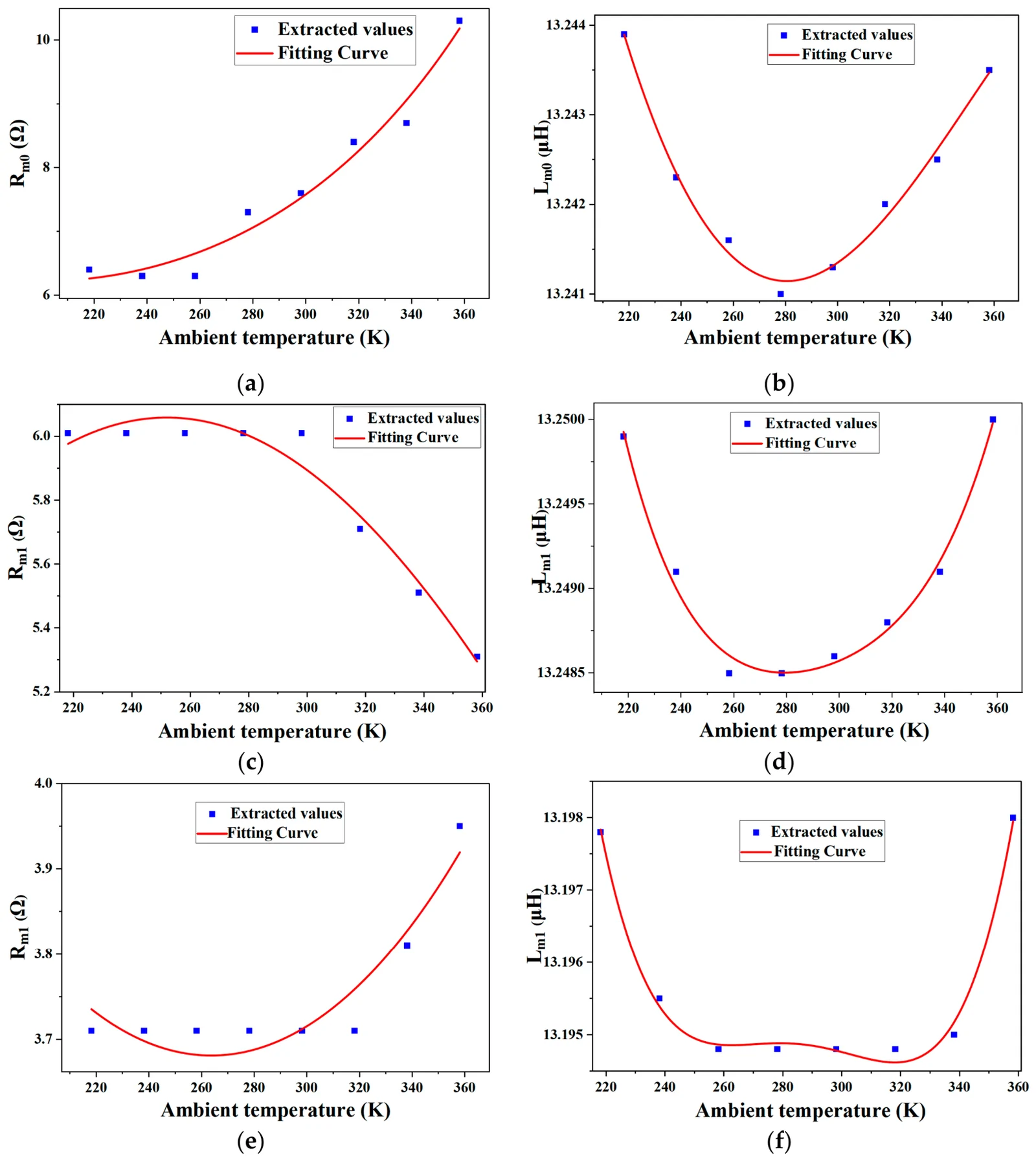
Extracted temperature-dependent parameters in the proposed model and their fitting results: (**a**) *R_m_*_0_; (**b**) *L_m_*_0_; (**c**) *R_m_*_1_; (**d**) *L_m_*_1_; (**e**) *R_m_*_2_; (**f**) *L_m_*_2_.

The measurement results show that the temperature dependence of the resonant frequency is non-monotonic with the temperature increase from −55 °C to 85 °C. All the resonant frequencies have a similar trend, which is that the resonant frequency first increases and then decreases over the full measurement ambient temperature range. A similar case can be found in the reference [23]. The ranges of the y-axis are the same, which is equal to 0.25 MHz. Thus, it can analyze the change in the resonant frequencies of different Modes with the temperature-dependent parameters. When comparing the results in Figure 6a,b, the spurious mode 1 has less variation than the main mode based on the model. The change is larger when ambient temperatures deviate far from room temperature. What is more, the results shown in Figure 6c indicate that the resonant frequencies almost do not change when the ambient temperature varies from −35 °C to 55 °C. It is shown that spurious mode 2 is less influential, but massive changes in the resonant frequency occur when the ambient temperature varies from −55 °C to 85 °C. This can be seen in Figure 6d. The reason may be that the amplitude of the spurious modes is smaller than that of the main mode, and the parameter values of the subcircuit for the spurious modes change little. The temperature-dependent parameters are contained in the proposed model and are shown in Figure 2b. They have the values at different temperatures in Figure 7.

With the temperature-dependent parameters, the proposed model can accurately describe the effect of ambient temperature on RF performance, which is significant in RF SAW filter designs. The inductance parameters *L_m_*_0_, *L_m_*_1_, and *L_m_*_2_ have some relationship with the results in Figure 6, which is reasonable that the resonant frequencies are adjusted with the inductance parameters. All inductance values show a similar trend: they first decrease, then increase over the full ambient temperature measurement range. The resistance parameters *R_m_*_0_, *R_m_*_1_, and *R_m_*_2_ are monotonous with the increase in temperature, which is interesting. As described above, the extracted parameters of the proposed model at different ambient temperatures are shown in Figure 7. All the values appear temperature-dependent, although some of the values are almost constant with the change in the ambient temperature. To obtain an SAW resonator model in the temperature-dependent range, the modeling processes need to be carried out. The results are presented in Figure 7. The calculation process for the temperature-dependent parameters in Figure 2b can be obtained with the following Expressions (3) and (4).

(3) $$R_{mi}\left(T\right)=e^{\left(R_{i1}+R_{i2}T+R_{i3}T^{2}\right)},i=0,1,2,$$

(4) $$L_{mi}\left(T\right)=L_{i0}+L_{i1}T+L_{i2}T^{2}+L_{i3}T^{3}+L_{i4}T^{4},i=0,1,2,$$

where *T* is the ambient temperature in K. *R*_i1_, *R*_i2_, and *R*_i3_ are the fitting parameters for the resistance *R_m_*_i_. *L_i_*_0_, *L_i_*_1_, *L_i_*_2_, *L_i_*_3_, and *L_i_*_4_ are the fitting parameters for the inductance *L_mi_*. The parameter values in Expressions (3) and (4) are shown in Table 3.

## 3. Results and Discussion

**Figure 8:**
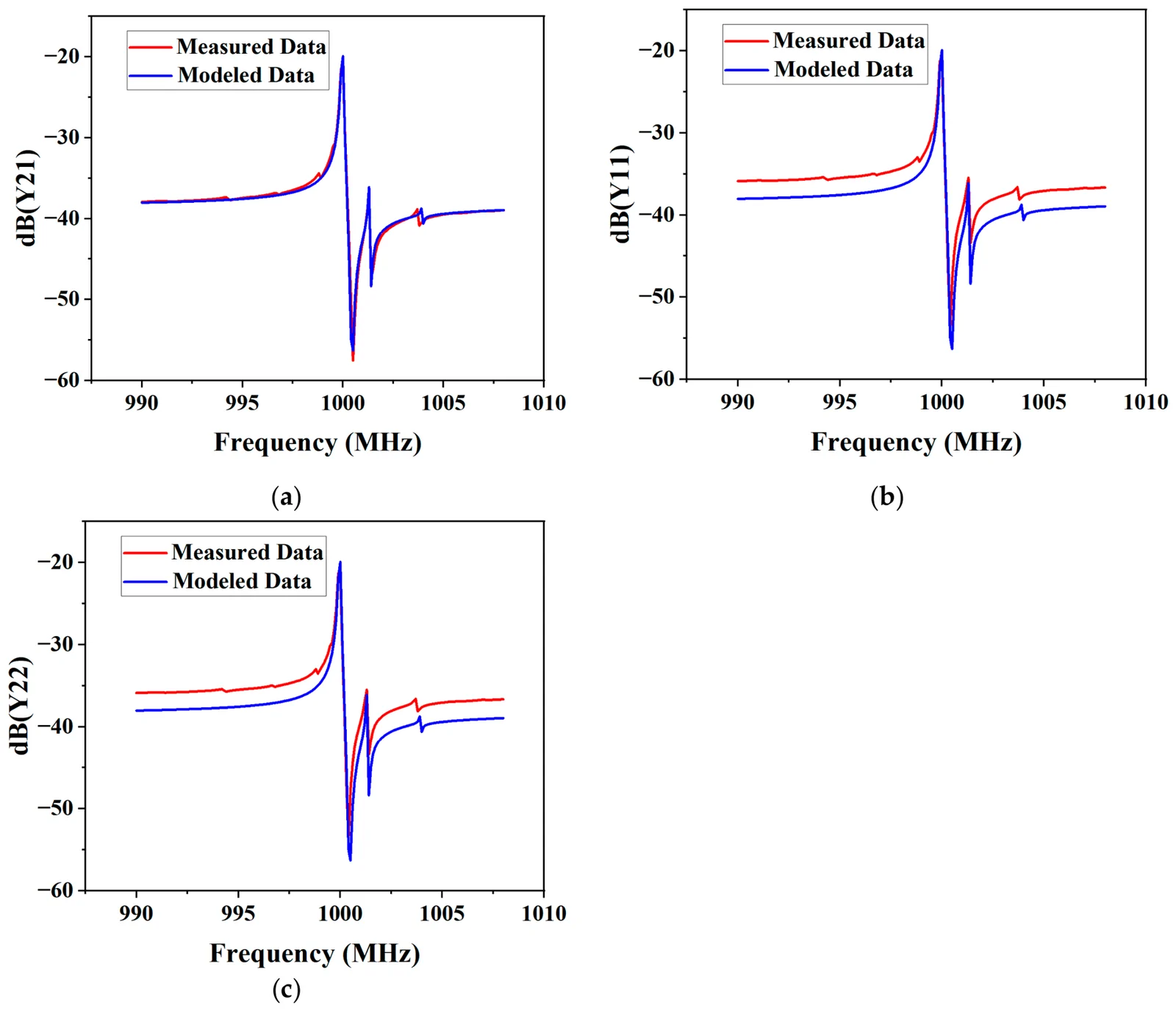
Comparison between measured and simulated Y-parameters of the SAW resonator at −55 °C: (**a**) Y_21_; (**b**) Y_11_; (**c**) Y_22_.

**Figure 9:**
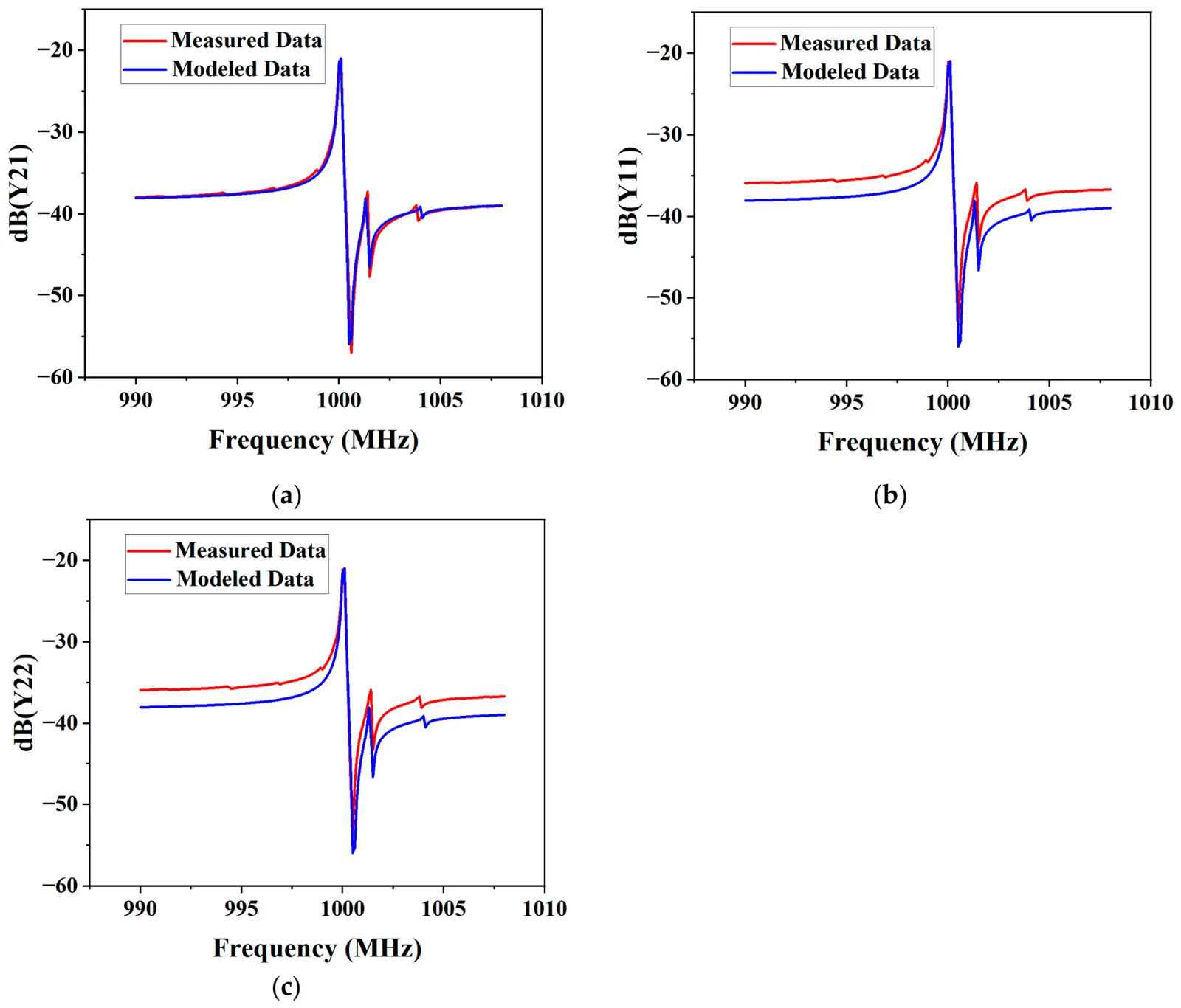
Comparison between measured and simulated Y-parameters of the SAW resonator at −15 °C: (**a**) Y_21_; (**b**) Y_11_; (**c**) Y_22_.

**Figure 10:**
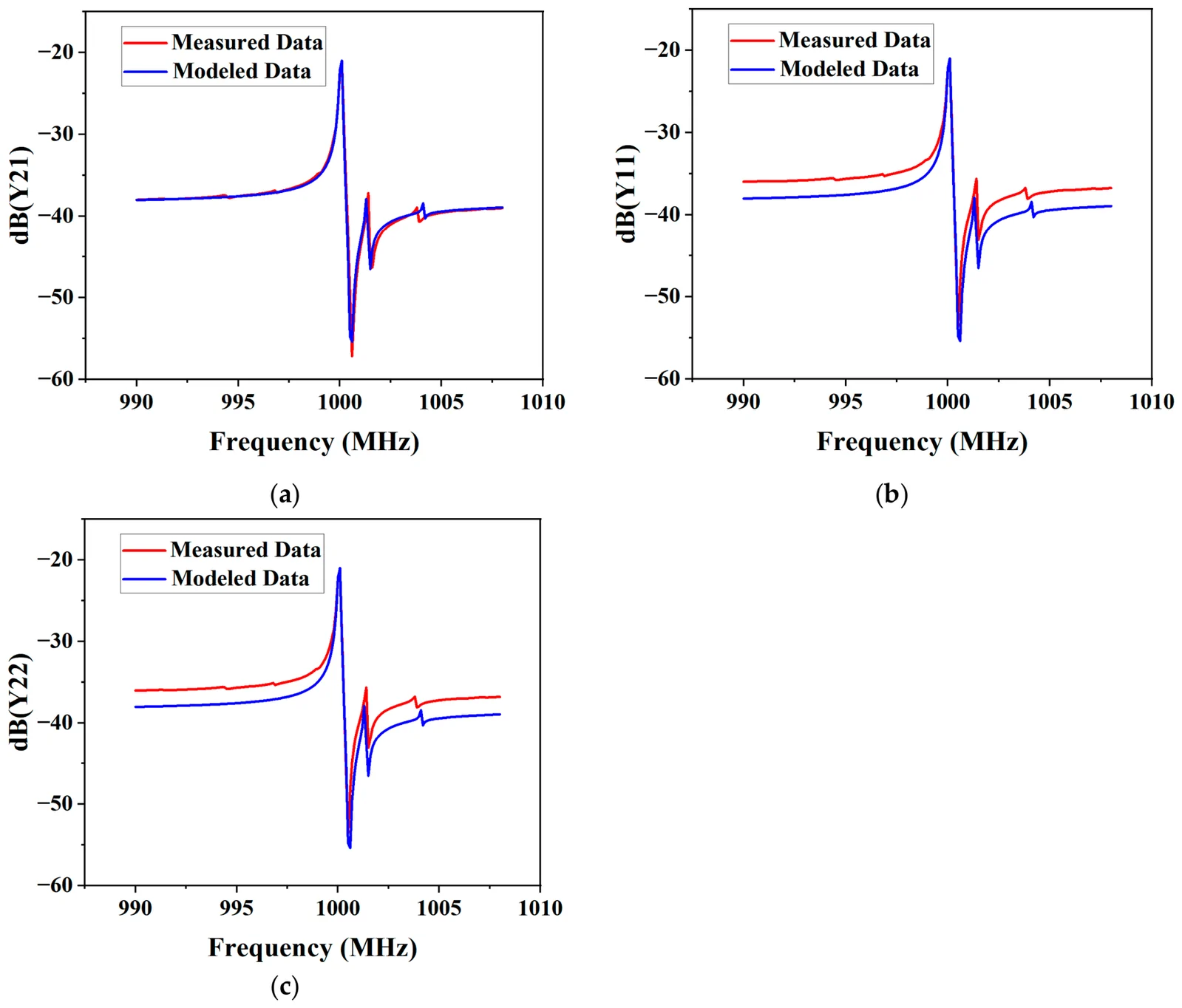
Comparison between measured and simulated Y-parameters of the SAW resonator at 25 °C: (**a**) Y_21_; (**b**) Y_11_; (**c**) Y_22_.

**Figure 11:**
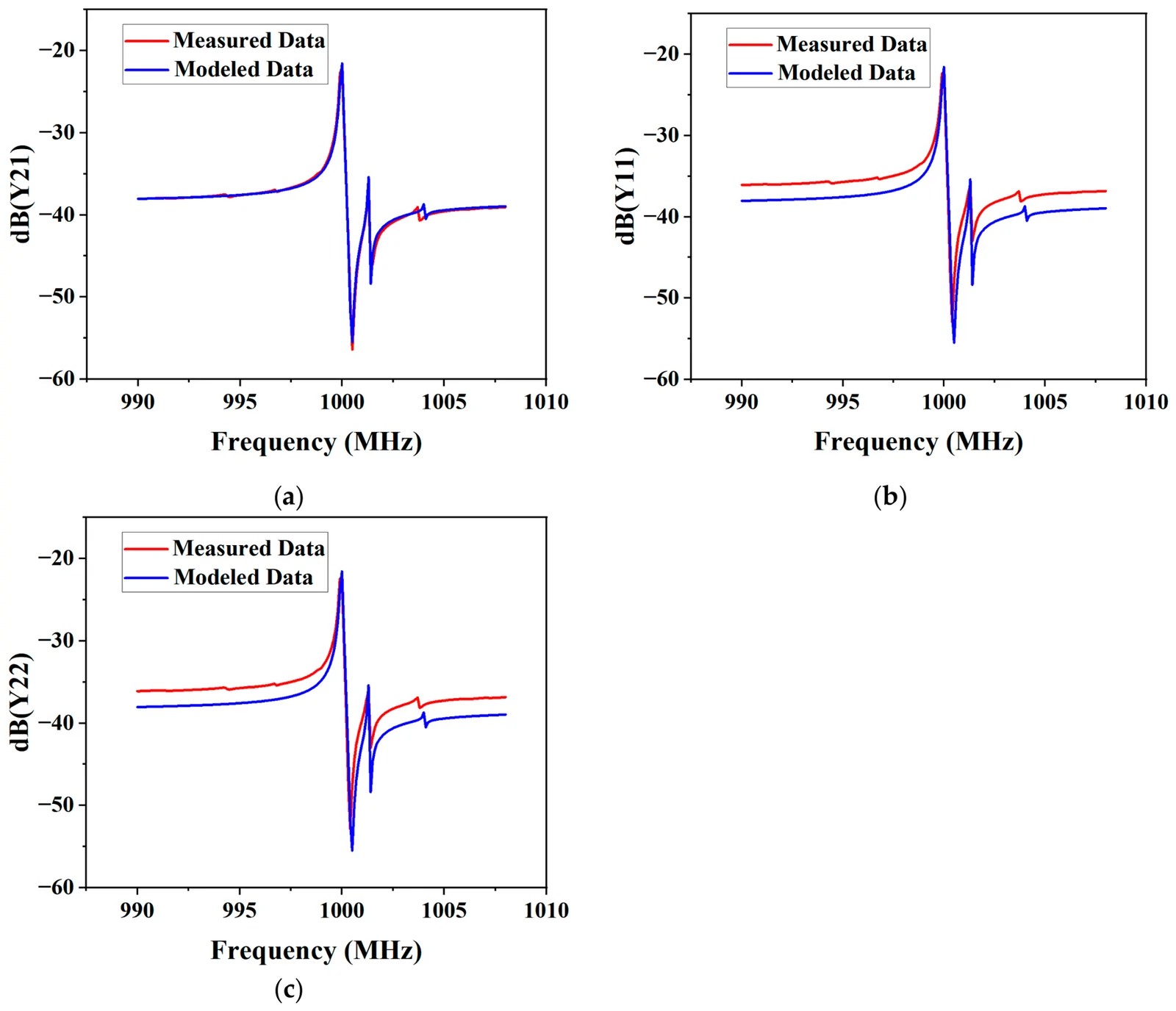
Comparison between measured and simulated Y-parameters of the SAW resonator at 85 °C: (**a**) Y_21_; (**b**) Y_11_; (**c**) Y_22_.

The temperature-dependent model for the SAW resonator is verified with temperature-dependent testing. The temperature range is from −55 °C to 85 °C. The DUT is measured after the chamber temperature has stabilized for 180s. The Y-parameters are obtained at each temperature set point using the VNA and the proposed model. For the modeling verification, the temperature-dependent parameters in Section 2.2 of the proposed model are employed, which are implemented in the equivalent circuits and simulated using the ADS software in order to obtain the Y-parameters of the proposed model for each measurement temperature. With the simulation results and the measurement results, the Y-parameter data of the SAW device are compared, which are shown in Figure 8, Figure 9, Figure 10 and Figure 11. The ambient temperatures −55 °C, −15 °C, 25 °C, and 85 °C are employed, which cover the full range and can indicate that the model is accurate. The parameters Y_21_, Y_11_, and Y_22_ are provided in Figure 8, Figure 9, Figure 10 and Figure 11.

The results in Figure 8 show the verification under the ambient temperature of −55 °C, which shows that the simulations are in very good agreement with the experimental results. For the main mode and spurious mode 1, the comparison results indicate that the proposed model can accurately describe the resonance frequency and its RF transmission characteristics. There are some errors in the Y_11_ and Y_22_ parameters, which can also be found in the paper [23], and these parameters reflect the return loss. The error function applied is defined as Expression (5). The fitting errors are 0.79%, 6.23%, and 6.23% for the Y_21_, Y_11_, and Y_22_ parameters, respectively.

(5) $$\varepsilon =\frac{1}{N}\sum_{n=1}^{N}\frac{\left|Y_{mea}\left(i\right)-Y_{sim}\left(i\right)\right|}{\left|Y_{mea}\left(i\right)\right|}$$

where *Y_mea_* and *Y_sim_* are measured and simulated data, and N is the number of sample points.

For the Y-parameters, the validation of the model under the ambient temperatures of −15 °C, 25 °C, and 85 °C is proposed as follows:

As can be seen from Figure 9, Figure 10 and Figure 11, the simulations are in good agreement with the tested data, especially for the frequency near the resonance frequency of the main mode. All the figures indicate that the proposed model is valid for all the temperatures, including the extreme ambient temperature. For the test temperature of −15 °C, the fitting errors are 0.84%, 6.19%, and 6.14% for the Y_21_, Y_11_, and Y_22_ parameters, respectively. The fitting errors for the measurement temperature of 25 °C are 0.84%, 5.78%, and 5.68% for the Y_21_, Y_11_, and Y_22_ parameters, respectively. Finally, the fitting errors for the measurement temperature of 85 °C are 0.56%, 5.75%, and 5.70% for the Y_21_, Y_11_, and Y_22_ parameters, respectively. The proposed results can describe the behavior of a two-port SAW resonator very well under temperature-dependent conditions, which proves that the proposed modeling process of the SAW device is good. The proposed model can be employed to design an RF filter in a wireless communication system.

## 4. Conclusions

The equivalent circuit modeling process for a SAW resonator with spurious modes under temperature-dependent conditions is proposed in this paper. Different from the reported paper, the isolation capacitance is proposed in this paper to address the resonance frequency of different modes, especially when their frequencies are very close. The SAW devices are tested under temperature-dependent environments, which shows that the resonance frequencies of the spurious mode are less influenced when the temperatures are near room temperature, but are largely influenced by the extreme ambient temperatures. It is investigated with the temperature-dependent parameters in the model, which are proposed in the paper. In the end, the measurement data of the SAW resonators are modeled with temperature-dependent parameters of the proposed model. The errors between the measured and modeled data at all temperature ranges are less than 0.84%, 6.23%, and 6.23% for the Y_21_, Y_11_, and Y_22_ parameters, respectively. This indicates that the model is accurate and the modeling process is effective. The proposed work is important for SAW device investigation and designing RF filters in wireless communication.

## Figures and Tables

**Table 1:** Parameters in the ECM of the commercial SAW resonator.

Description	Rm (Ohm)	Rs (Ohm)	Lm (μH)	Cm (fF)	C_0_ (pF)
From Datasheet	10.7	Na	15.3	1.66	2.75
Extracted	7.6	2.1	13.241	1.9127	1.948

**Table 2:** Parameters in the ECM of the commercial SAW resonator with spurious response.

Mode	C_0_ (pF)	R_m_ (Ohm)	L_m_ (μH)	C_m_ (fF)	f_s_ (MHz)
0	1.198	7.6	13.241	1.9127	1000.084
1	2.3	6.01	13.2486	1.9076	1001.132
2	0.8	3.71	13.1948	1.908	1003.066

**Table 3:** Physical parameters in Expressions (3) and (4).

Parameters	Values	Parameters	Values
*R*_01_	2.604	*R*_02_	−0.008
*R*_03_	1.955 × 10^−5^	*R*_11_	1.042
*R*_12_	0.006	*R*_13_	−1.195 × 10^−5^
*R*_21_	1.792	*R*_22_	−0.0037
*R*_23_	0.703 × 10^−5^	*L*_00_	13.311
*L*_01_	−4.91 × 10^−4^	*L*_02_	5.1416 × 10^−7^
*L*_03_	2.408 × 10^−9^	*L*_04_	−4.143 × 10^−12^
*L*_10_	13.465	*L*_11_	−2.88 × 10^−3^
*L*_12_	1.448 × 10^−5^	*L*_13_	−3.260 × 10^−8^
*L*_14_	2.782 × 10^−11^	*L*_20_	14.435
*L*_21_	−0.0175	*L*_22_	9.210 × 10^−5^
*L*_23_	−2.15 × 10^−7^	*L*_24_	1.876 × 10^−10^

## Data Availability

The research data will be provided upon approval by the author.
